# A Method for Temporally Resolved Continuous Inline Measurement of Multiple Solute Concentrations With Microfluidic Spectroscopy

**DOI:** 10.1109/OJEMB.2025.3555807

**Published:** 2025-03-28

**Authors:** Andrea Lorenzo Henri Sergio Detry, Vinny Chandran Suja, Nathaniel Merriman Sims, Robert A. Peterfreund, David E. Arney

**Affiliations:** Department of Chemical, Materials, Industrial Production EngineeringUniversity of Naples Federico II 9307 80125 Naples Italy; Massachusetts General Hospital, Department of AnesthesiaCritical Care, Pain Medicine Boston MA 02114 USA; School of Engineering, Applied SciencesHarvard University 1812 Cambridge MA 02134 USA; Wyss Institute for Biologically Inspired Engineering, 201 Brookline Ave 465574 Boston MA 02138 USA; Massachusetts General Hospital, Department of AnesthesiaCritical Care, Pain Medicine Boston MA 02114 USA

**Keywords:** Inline measurements, microfluidics, multi-fluid analysis, real-time monitoring, spectroscopy

## Abstract

*Goal:* To develop a compact, real-time microfluidic spectroscopy system capable of simultaneously measuring the concentrations of multiple solutes flowing together through a single fluid pathway with high temporal resolution. *Methods:* The measurement system integrates a Z-flow cell and dual-wavelength LED light sources with a compact spectrophotometer. The experimental setup consisted of two clinical infusion pumps delivering distinct marker dyes through a common fluid pathway composed of a clinical manifold and a single lumen of a clinical intravascular catheter, while a third pump delivered an inert carrier fluid. Concentration measurements of the mixed dyes were performed at high-frequency sampling intervals, with dynamic pump rate adjustments to evaluate the system's ability to detect real-time changes in solute concentration. A MATLAB-based control application enabled automated data acquisition, processing, and system control to enhance experimental efficiency. *Results:* The system accurately measured solute concentrations, capturing temporal variations with high precision. It demonstrated high reproducibility with a standard error of the mean no larger than $0.19 \,\mu \mathrm{g}\mathrm{/}\mathrm{m}\mathrm{L}$ for Erioglaucine and $1.32 \,\mu \mathrm{g}\mathrm{/}\mathrm{m}\mathrm{L}$ for Tartrazine at steady state, and high accuracy with a maximum deviation of $0.21 \,\mu \mathrm{g}\mathrm{/}\mathrm{m}\mathrm{L}$ for Erioglaucine and $0.5 \,\mu \mathrm{g}\mathrm{/}\mathrm{m}\mathrm{L}$ for Tartrazine from the expected steady-state concentrations. *Conclusions:* This system enables continuous, real-time monitoring of multiple solutes in dynamic flow conditions, offering a portable solution with high sensitivity to temporal concentration changes—advancing beyond traditional static fluid measurement methods.

## I. Introduction

Accurate and sensitive measurement of solute concentration in flowing liquids is crucial in several fields. Time-resolved measurements of drug concentrations in multi-drug infusion systems are pivotal for ongoing efforts focused on improving the dosing accuracy and delivery kinetics of critical care medications [1], [2], [3]. For example, translating the recent advancements in infusion management, such as physics-informed reinforcement learning approaches [4], is heavily reliant on high-fidelity techniques for continuous drug concentration measurements. In industrial settings, real-time monitoring of fluid concentrations can significantly improve process control, enhancing consistency and safety while reducing waste [5], [6]. For example, in continuous flow synthesis, the ability to monitor and adjust reactant concentrations in real-time can optimize reaction yields and product quality [7], [8].

Conventional techniques for obtaining time-resolved species concentrations often require bulky equipment such as fraction collectors [9] and batch processing, limiting their temporal resolution and applicability in real-time point-of-care settings. To address these limitations, researchers are increasingly exploring compact platforms. A particularly attractive novel method leverages microfluidic spectroscopy. By employing a compact arrangement of flow cells, multi-spectral illuminators, and spectrophotometers, this method makes it possible to non-invasively monitor the concentration of target analytes in real time by measuring the spectral signature of a flowing liquid. The compact and portable nature of this method makes it suitable for field applications in remote or hazardous locations, providing critical data where traditional methods are impractical [10], [11]. The adaptability of this method also supports its use in automated laboratories for drug discovery and materials science, where precise control and monitoring of fluidic processes are essential [12], [13].

Microscopic spectroscopy has been explored for various applications, including performance evaluations of clinical infusion pumps [14], [15], [16]. However, most studies have been constrained to a limited set of fluids, and often rely on a single dye as a trackable marker.Additionally, comprehensive methodological guidance remains scarce, limiting the reproducibility and broader adoption of these approaches. Existing research has demonstrated the feasibility of spectroscopic analysis in microfluidic platforms and digital microfluidic devices [17], yet few studies provide detailed procedural insights necessary for reliable replication and adaptation. In clinical settings, real-time monitoring of multiple drug infusions remains a challenge. Studies have highlighted the complexities of intravenous drug delivery, particularly in neonates, where achieving precise dosing is critical [18], [19]. Conventional spectrophotometric approaches have been used to analyze drug delivery dynamics [20] in laboratory models, but these rely on batch sampling rather than real-time, inline measurements. The lack of continuous monitoring methods underscores the need for advanced techniques that enable high-frequency, multi-fluid analysis with minimal disruption to ongoing infusion processes.

The present study proposes a refined approach to microfluidic spectroscopy for real-time inline fluid concentration measurement, addressing the identified gaps in the existing literature. While similar systems exist, [1], [14], [15], [16] detailed systematic characterization and comprehensive methodological guidelines remain limited. The proposed system is able to simultaneously analyze multiple fluids within microchannels using a compact and portable setup comprising a Z-flow cell, dual-wavelength LEDs, and a spectrophotometer. The paper further describes the development of a user-friendly MATLAB application to control pumps, acquire data from the spectrophotometer, and perform concentration calculations. By providing detailed descriptions of measurement principles and experimental configurations, we aim to bridge the accessibility gap for researchers seeking to replicate and adapt such systems. This comprehensive approach facilitates broader adoption and application in diverse biomedical, chemical, and environmental contexts. In conclusion, this study provides a comprehensive explanation of the methodology and practical guidance for replication and adaptation, thus going beyond existing literature. The creation of an open-source MATLAB application provides a readily accessible platform for researchers, thereby facilitating the adoption of this technique on a broader scale. The combination of precision, portability, and multi-fluid analysis capabilities represented in this work serves to address significant gaps in current methodologies and lays the foundation for advanced applications in biomedical, chemical, and environmental domains.

## II. Materials and Methods

### A. Clinically Relevant Experimental Framework

**Fig. 1.**
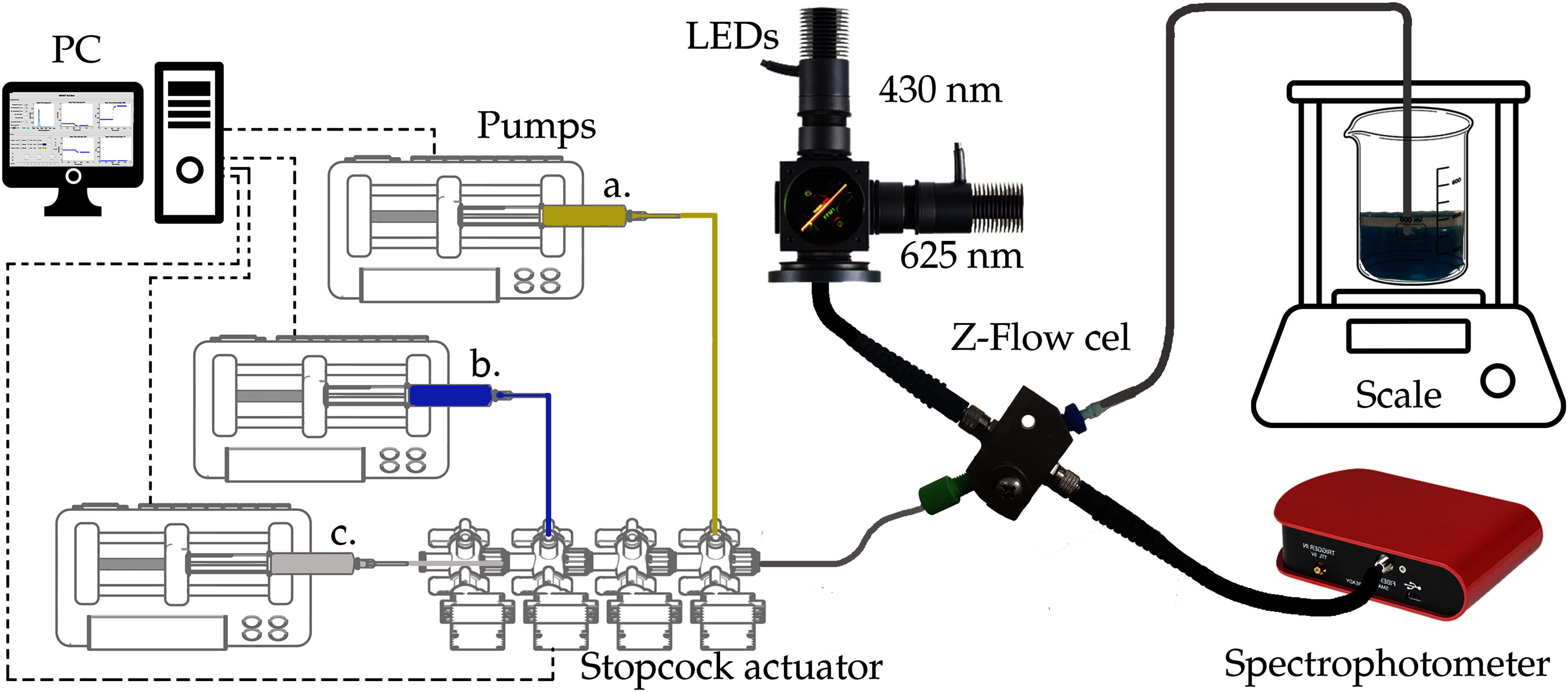
Integrated setup of the microfluidic spectroscopy system; at left a computer with the Matlab App that controls the pump arrangement and the stopcock actuator, at left the LED light sources at 430 nm and 625 nm, the Z-Flow cell for fluid analysis, the spectrophotometer, and a precision scale.

To demonstrate the microfluidic spectroscopy technique, we recreate a clinically relevant scenario prevalent in both critical care and anesthesia management. As shown in Fig. 1, in this scenario we have multiple drugs enter a common fluid pathway via a manifold for delivery to the patient's circulation via continuous central venous infusion. A commonly used infusion system includes a syringe pump driving the flow of a physiologically inert carrier fluid such as normal saline (NaCl 0.9% w/v). The carrier then flows through a manifold consisting of 4 standard stopcocks arranged linearly. The manifold connects to a central venous catheter through which fluid flows into circulation.

In our study, we employed food-grade dyes with physical properties comparable to those of actual drugs to emulate clinical infusion scenarios. This approach allows us to safely and effectively simulate the behavior of drug mixtures in a controlled laboratory setting, ensuring that our findings are relevant to real-world medical applications.

For demonstration, here we use three pumps linked to color-coded syringes: Yellow (Fig. 1(a)) for Tartrazine, Blue (Fig. 1(b)) for Erioglaucine disodium salt, and Gray (Fig. 1(c)) for the Saline carrier. The decision to use two food-grade dyes, Erioglaucine disodium salt (Sigma Aldrich, catalog number 861146-25 G) and Tartrazine (Sigma Aldrich, catalog number T0388-100 G), was based on the significant difference in their absorption wavelengths, as previously demonstrated by Tsao et al. [3]. These dyes effectively model the infusion of actual drugs, providing a reliable basis for validating our method's accuracy in clinical applications.

### B. Pumps

To infuse the fluid in this study, we used the LEGATO 110 SYRINGE PUMP from (kdScientific, USA) [21]. These pumps can be interfaced to a computer via a serial port for remotely controlling the flow rate in real time. The pumps were fitted with BD 50 ml syringes with Luer Lock tips and connected to a stopcock assembly using BC 560 tubing.

### C. Stopcock Assembly and Servo Actuator

**Fig. 2.**
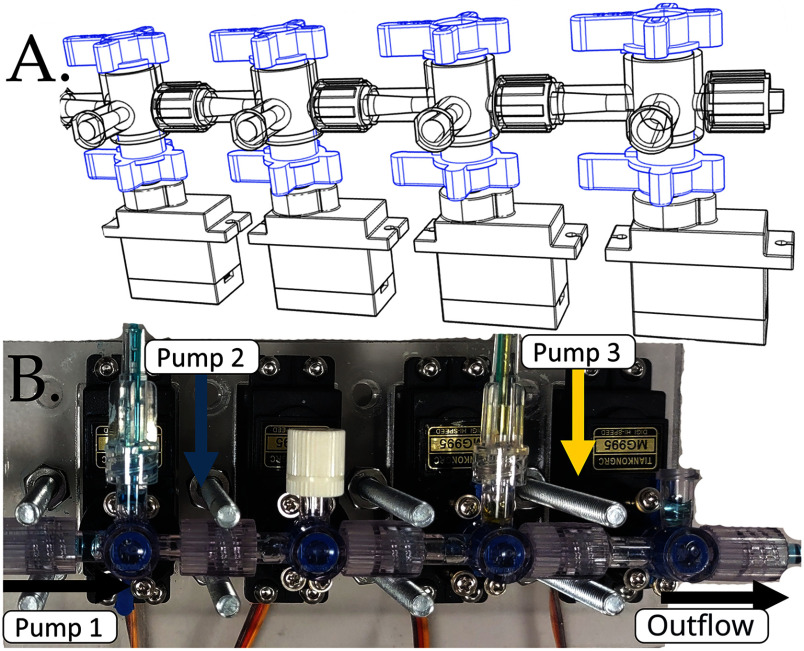
Stopcock assembly design and implementation; (a) CAD rendering showcasing the setup of servomotors mounted on stopcocks, and (b) a photograph of the actual assembled system featuring the stopcocks in operation.

Fig. 2 shows the stopcock assembly, made with four commercially available stopcocks connected to four servomotors [22] that are able to control the direction of flow through the stopcock. An Arduino Uno Rev3 [23] acts as the processor for controlling the servo motors; it is connected to a power supply and programmed to read commands sent via the serial port and translate them into commands for individual servo motors. For example, the computer could send a command through the serial port as follows: A180,B90,C0,D270. In this example, the first servo (called ‘A’) will be positioned at $180^{\circ }$, the second (‘B’) at $90^{\circ }$, the third (‘C’) at $0^{\circ }$, and the fourth (‘D’) at $270^{\circ }$.

### D. Flow Cell

**Fig. 3.**
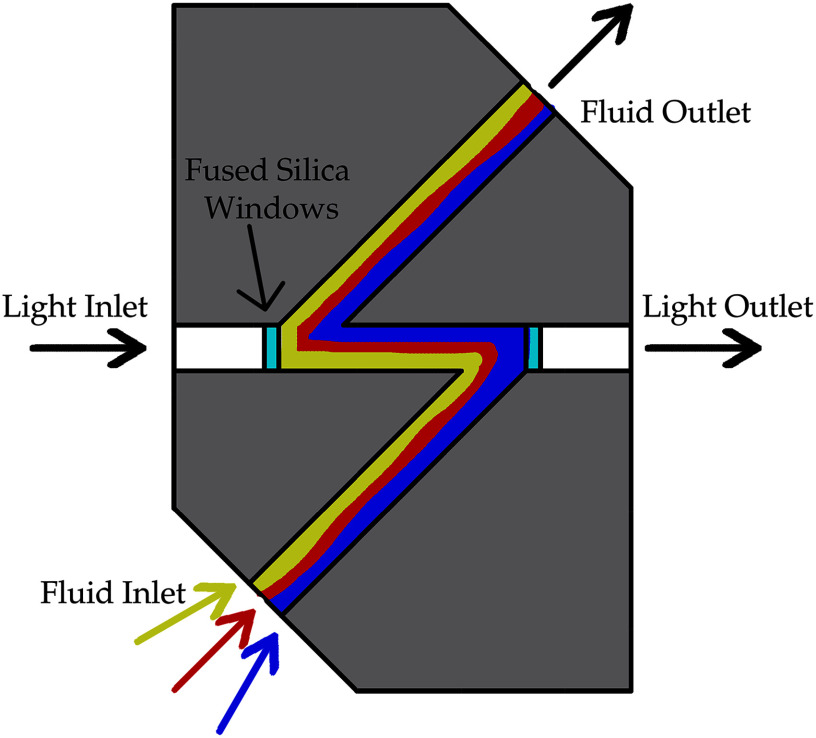
Schematic diagram of an ultra-low volume Z-flow cell employed in our setup.

The Z-flow cell [24], as schematically shown in Fig. 3, is a commercially available component of our microfluidic spectroscopy system (Ocean Optics #FIA-ZSMA-ML-2.5-PE). This device is specifically designed for flow injection analysis (FIA) and is constructed from stainless steel. The selected Z-flow cell has an internal volume of 26 $\mu \mathrm{L}$ and a path length of 10 $\mathrm{m}\mathrm{m}$, operating across a wavelength range from 210 $\mathrm{n}\mathrm{m}$ to 2 $\mu \mathrm{m}$, making it ideal for high sensitivity and precision optical absorbance measurements.

The Z-design of the flow cell offers several advantages over these other designs such as straight through and serpentine configuration. Its configuration maximizes the optical path length while maintaining a low internal volume, which is crucial for high sensitivity measurements with minimal sample usage. This design ensures that the entire sample is exposed to the light path, improving the accuracy and consistency of absorbance measurements. Additionally, the Z-flow cell minimizes additional dead volume, reducing the amount of sample required and ensuring better temporal correlation between the measured concentration and the actual concentration at the intended location (in our case at the tip of central venous catheter). Overall, the Z-flow cell combines the benefits of a long optical path length and low internal volume, making it an optimal choice for precise and efficient fluid analysis in various scientific and industrial applications [25], [26], [27].

### E. Light Source

**Fig. 4.**
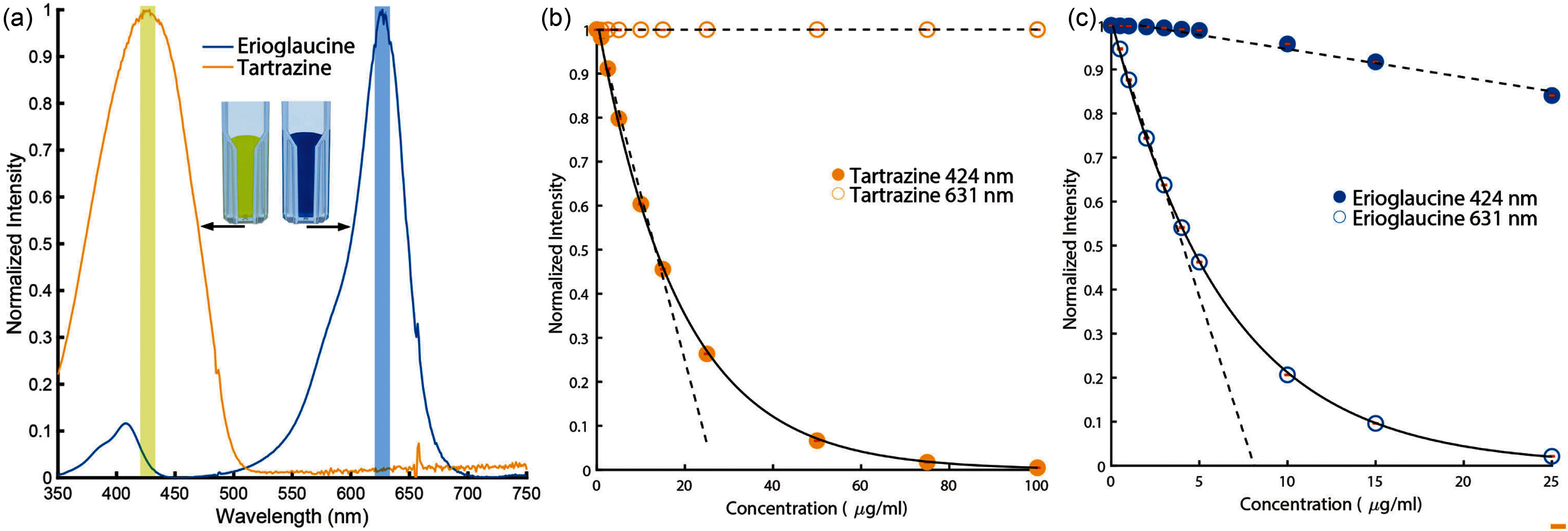
(a) Absorption spectrum of Erioglaucine Blue and Tartrazine. Concentration versus normalized transmission intensity calibration curves for Erioglaucine Blue (b) and Tartrazine (c) Solid curves correspond to single exponential fits while dashed lines correspond to linear fits.

The light source depicted in Fig. 1 utilizes two high-intensity LEDs as its primary illumination source. The first LED, the Thorlabs M625L4 [28], emits light at a peak wavelength of 625 nm, while the second, the Thorlabs M430L4 [29], operates at peak wavelength of 430 nm. These LEDs were specifically chosen as their peak emission closely matches the absorption peak of our dyes (Fig. 4(a)). Both LEDs are integrated into our system using a Thorlabs C4W 30 mm Cage Cube [30], which includes a dichroic mirror – the Thorlabs DMLP505R [31] – and a specialized lens – the Thorlabs LA4647 [32]. This lens is able to converge the light beams from both LEDs into a single, collimated beam, ensuring a uniform beam in the inlet optical fiber cable that is connected to the flow cell.

The LEDs are powered and controlled by the Thorlabs LEDD1B T-Cube LED Driver [33], allowing for precise modulation of light intensity. This driver is connected to a KPS201 Power Supply Unit [34], which delivers a stable 15 V, 2.66 A output, ensuring consistent illumination. While the current configuration employs two light sources, the system can be readily scaled to incorporate additional LEDs operating at different wavelengths for multiplexed measurements of 3 or more solutes. This flexibility allows for the analysis of solutes with absorption spectra having peaks distinct from 625 nm and 430 nm, thus expanding the system's applicability for the simultaneous measurement of more than two solutes.

### F. Spectrophotometer

The core of our spectrophotometric assembly is the Thorlabs CCS100 Compact spectrophotometer [35], depicted in Fig. 1, which can analyze spectral intensities over a range of wavelengths spanning from $350 \,\mathrm{n}\mathrm{m}$ to $700 \,\mathrm{n}\mathrm{m}$ with a resolution of less than $0.5 \,\mathrm{n}\mathrm{m}$ Full Width at Half Maximum (FWHM) at $435 \,\mathrm{n}\mathrm{m}$ and less than $0.6 \,\mathrm{n}\mathrm{m}$ FWHM at $633 \,\mathrm{n}\mathrm{m}$. This range is particularly well-suited for our needs, as it encompasses the emission wavelengths of our LED light sources. The CCS100 also features an adjustable integration time window (from $10 \,\mu \mathrm{s}$ to $60 \,\mathrm{s}$), allowing a suitable balance between acquisition frequency and signal-to-noise ratio. To convert spectral intensity data into solute concentrations, a calibration procedure was performed using known mixtures of Erioglaucine and Tartrazine. The intensity readings at two selected wavelengths (424 nm and 631 nm) were used in combination with calibration curves to recover dye concentrations in real time. Details of the calibration process, equations used, and corresponding calibration curves can be found in the Supplementary Materials and Fig. 4.

### G. Analytical Balance

An analytical balance (Ainsworth M-220) was employed to measure the total infused fluid volume over time (Fig. 1). This information was used to orthogonally validate the total infused mass flow rate as a function of time. The selected M-220 model offers a sensitivity of 0.01 mg and a capacity of up to 220 g, aligning perfectly with the experimental requirements for accurately monitoring the relevant fluid mass flow rates in real-time.

### H. Graphical User Interface (GUI)

**Fig. 5.**
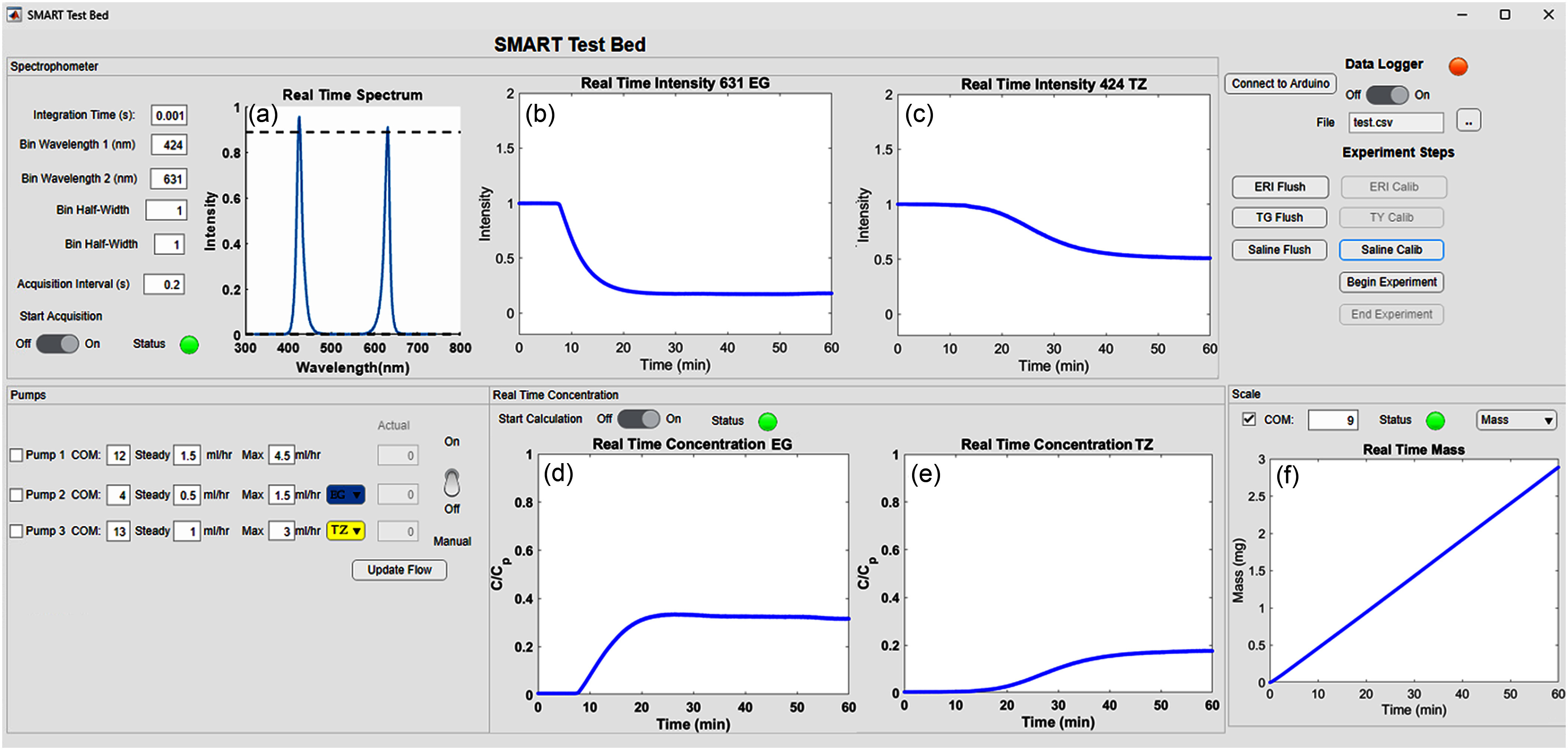
Interface of the MATLAB Application with representative data expected from a typical experiment. Panel (a) displays the real-time spectrum. Panels (b) and (c) show the real-time intensity plots for Erioglaucine (EG) at 631 nm, the absorption peak for EG (b) and for Tartrazine (TZ) at 424 nm, the absorption peak for TZ (c). The decrease in intensity observed in these panels corresponds to the increase in dye concentration. Panels (d) and (e) show the real-time concentration ($C$) of Erioglaucine and Tartrazine, respectively, normalized as $C/C_{0}$, where $C_{0}$ is the concentration of the dye in the syringe (or equivalently the maximum possible concentration). Panel (f) presents the real-time mass measurement from the scale. The spectrophotometer section (top left), the pumps section (bottom left), the real-time concentration graphs (bottom center), the Data Logger section (top right), and the analytical balance/scale section (bottom right) complete the interface layout.

To facilitate user control and real-time data acquisition, we also developed a standalone MATLAB application with an intuitive graphical user interface (see Fig. 5). The GUI enables synchronized control of spectrophotometer acquisition, syringe pumps, stopcock actuators, and the analytical balance, while providing real-time plots of solute concentrations and infusion mass. A detailed description of the GUI features, along with the full interface layout, is provided in the Supplementary Materials.

## III. Results

**Fig. 6.**
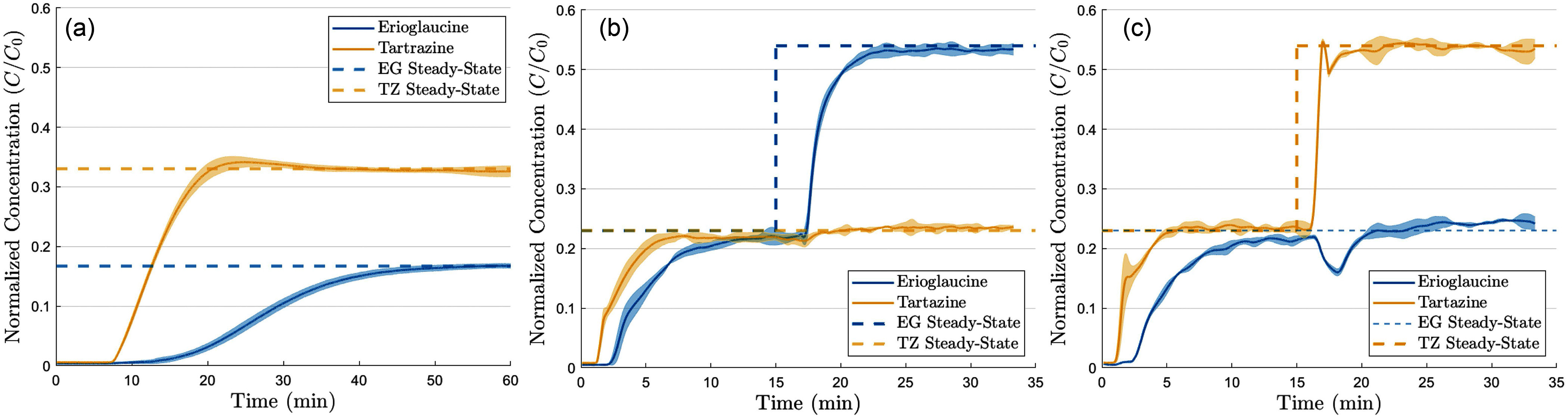
Panels (a), (b), and (c) show the measured normalized dye concentration ($C/C_{0}$) as a function of time. $C/C_{0}$ is obtained by normalizing the measured instantaneous dye concentration ($C$) with the dye concentration in the syringe ($C_{0}$). The shaded error bars indicate the standard error of the mean obtained across three independent trials. The infusions were started at time zero with pumps initially off and all the tubing filled with saline, resulting in an initial concentration of zero for both the dyes.

To validate the performance and reliability of our microfluidic spectroscopy method for in-line fluid concentration measurements, we conducted a series of experiments (Table I), the results of which are presented in Fig. 6(a), (b), and (c). For each condition, we performed three independent trials to assess the repeatability of our measurements. The data from these repeated experiments are represented as concentration versus time plots, with the shaded error bars indicating of the standard error of the mean (SEM)

**TABLE I:** Flow Rates in $\mathrm{m}\mathrm{L}\mathrm{h}^{-1}$ of Each Pump and the Expected Normalized Steady State Solute Concentrations ($C/C_{0}|_{t}\rightarrow \infty$) at the Measurement Point During Different Time Intervals for Validation Experiments **A**, **B**, and **C**

Exp.	Time	Pump 1	Pump 2	Pump 3	$C/C_{0} |_{t\rightarrow \infty }$	$C/C_{0} |_{t\rightarrow \infty }$
		(Saline)	(EG)	(TZ)	(EG)	(TZ)
a	(0$\mathrm{min}$ to 60$\mathrm{min}$)	1.5	0.5	1	0.167	0.33
b	(0$\mathrm{min}$ to 15$\mathrm{min}$)	7	3	3	0.23	0.23
	(15$\mathrm{min}$ to 35$\mathrm{min}$)	3	7	3	0.54	0.23
c	(0$\mathrm{min}$ to 15$\mathrm{min}$)	7	3	3	0.23	0.23
	(15$\mathrm{min}$ to 35$\mathrm{min}$)	3	3	7	0.23	0.54

Pump 1 Delivered Saline, While Pumps 2 and 3 Respectively Delivered Erioglaucine (EG) and Tartrazine (TZ) Solutions. To Obtain $C/C_{0}|_{t}\rightarrow \infty$, the Instantaneous Solute Concentration ($C$) is Normalized With the Concentration of the Dye in the Syringe ($C_{0}$), Which Respectively for Erioglaucine (EG) and Tartrazine (TZ) Solutions are $25 \mu \mathrm{g}\mathrm{/}\mathrm{m}\mathrm{L}$ and $100 \mu \mathrm{g}\mathrm{/}\mathrm{m}\mathrm{L}$

In Experiment **a** (Fig. 6(a)), the flow rates for all pumps were kept constant over the entire 60-minute interval, as outlined in Table I. This served as the baseline test, demonstrating the system's ability to measure concentration under steady flow conditions. The maximum error (SEM) of the concentration at steady state for Erioglaucine is $0.0559 \,\mu \mathrm{g}\mathrm{/}\mathrm{m}\mathrm{L}$, while for Tartrazine it is $0.5212 \,\mu \mathrm{g}\mathrm{/}\mathrm{m}\mathrm{L}$, highlighting the system's high precision under constant flow conditions.

In Experiment **b** (Fig. 6(b)), the flow rates of Pump 1 and Pump 2 were switched after 15 minutes of steady flow, with Pump 3 held constant. This experiment tests the system's adaptability to measure concentration under sudden changes in flow rates. In Experiment c (Fig. 6(c)), a similar switching event was implemented, but this time involving Pumps 1 and 3 after the initial 15-minute period. The maximum error (SEM) for Experiment **b** at steady state for Erioglaucine is ${0.1798 } \,\mu \mathrm{g}\mathrm{/}\mathrm{m}\mathrm{L}$, while for Tartrazine it is ${0.7893 } \,\mu \mathrm{g}\mathrm{/}\mathrm{m}\mathrm{L}$, for Experiment **c** Erioglaucine is ${0.1903 } \,\mu \mathrm{g}\mathrm{/}\mathrm{m}\mathrm{L}$, while for Tartrazine it is ${1.323 } \,\mu \mathrm{g}\mathrm{/}\mathrm{m}\mathrm{L}$.

The plots and the SEM values highlight the ability of the system to repeatably capture transient changes in concentration following changes in infusion rates, and demonstrate the system's reliability under dynamic conditions. It is important to note that the concentration of dyes approach the steady state with a finite time lag following a change in flow rate. This is due to the well-known dead-volume induced delays in propagating concentration changes to the measurement point [4], [36], and is not an artifact of the measurement technique.

Given an orthogonal measurement of the total flow rate at the measurement point, it is also possible to compute the concentration of a third non-absorbing solute exclusively injected via a third stream - in this case through the saline syringe. In our case, we obtain the instantaneous total flow rate as $\dot{Q_{Tot}} \approx \frac{\Delta mass}{\Delta t}$, where $\Delta mass$ is the change in mass recorded by the analytical balance between consecutive measurements, and $\Delta t$ is the change in time between those measurements. From conservation of mass in a compressible liquid, the total flow rate can be related to the flow rates of the individual streams as,

\begin{equation*}
\dot{Q_{\text{EG}}} + \dot{Q_{\text{TZ}}} + \dot{Q_{\text{Sal}}} = \dot{Q_{\text{Tot}}} \tag{1}
\end{equation*}

where $\dot{Q_{\text{EG}}}$ and $\dot{Q_{\text{TZ}}}$ are respectively the flow rates of Erioglaucine and Tartrazine, and $\dot{Q_{\text{Sal}}}$ is the flow rate of the saline stream. Rearranging the above equation we obtain,

\begin{equation*}
\frac{\dot{Q_{\text{Sal}}}}{\dot{Q_{\text{Tot}}}} \!=\! 1 - \frac{\dot{Q_{\text{EG}}}}{\dot{Q_{\text{Tot}}}} - \frac{\dot{Q_{\text{TZ}}}}{\dot{Q_{\text{Tot}}}} \Rightarrow \left. \frac{C}{C_{0}} \right|_{sal} = 1 - \left. \frac{C}{C_{0}} \right|_{EG} - \left. \frac{C}{C_{0}} \right|_{TZ} \tag{2}
\end{equation*}

Here the only unknown concentration is the normalized concentration of the species in the third stream ($C/C_{0}|_{sal}$). The above analysis also implies that given the total flow rate and the normalized concentration of two dyes, it is also possible to compute the individual instantaneous flow rates of all the three streams in the system. However, one should proceed with caution while inferring the above quantities from the measured dye concentrations. Specifically, the above analysis implicitly assumes that each of the solute is exclusively localized in one of the syringes, i.e, none of the syringe contains mixtures of solutes. Further, as is evident from Fig. 6, concentration of dyes approach the steady state with a finite time lag following a change in flow rate. This effect, originating from the well-understand dead-volume delays [4], [36], will drive transient and large inaccuracies in the computed flow rate until the measured dye concentrations approach their steady state values.

## IV. Discussion

This study demonstrates the detailed construction, feasibility, and reliability of microfluidic spectroscopy for continuously measuring solute concentrations within a flowing liquid stream. Section II details the construction of this compact platform with easily accessible components. Validation experiments, as detailed in Fig. 6, confirm that the system provides consistently accurate and repeatable measurements. The accuracy of the system was assessed by calculating the maximum absolute difference between the expected steady-state concentration values and the mean measured values, while repeatability was reported as the standard error of the mean. The results, summarized in Table II, show that the accuracy and repeatability for both Erioglaucine and Tartrazine remain within acceptable limits across different experimental conditions.

**TABLE II:** Summary of Accuracy and Repeatability of the Measurements

Experiment	Accuracy ($\mu \mathrm{g}\mathrm{/}\mathrm{m}\mathrm{L}$)		Repeatability ($\mu \mathrm{g}\mathrm{/}\mathrm{m}\mathrm{L}$)	
	EG	TZ	EG	TZ
EXP a	0.0131	0.2603	0.0559	0.5212
EXP b	0.1834	0.4984	0.1798	0.7893
EXP c	0.2124	0.2679	0.1903	1.323

Accuracy is Calculated as the Maximum Absolute Difference Between the Expected Steady-State Concentration and the Measured Mean Value. Repeatability is Evaluated as the SEM of the Measured Concentration Over Repeated Trials. Concentrations are Expressed in $\mu \mathrm{g}\mathrm{/}\mathrm{m}\mathrm{L}$

To facilitate the convenient adoption of this technology, we also developed a stand-alone MATLAB application with an intuitive graphical user interface. This application serves a central hub for conveniently controlling all features of the microfluidic spectroscopy platform including acquiring, processing and exporting time-resolved concentration data at high temporal resolution (maximum measurement frequency of 0.2 s). This software has been made publicly available to support further research and practical applications. Researchers and practitioners can access the code repository at https://github.com/andrea199/MatlabSpectroscopy, enabling further innovation and the broader implementation of this methodology across various domains.

Our methodology has several advantages over traditional techniques, including enhanced compactness, portability, and real-time monitoring capabilities. These attributes make it suitable for on-the-go applications and the development of closed-loop control systems, which are increasingly in demand in fields ranging from biomedicine to environmental science. These capabilities are also crucial for translating emerging technologies for drug infusion management such as the recently reported SMART (**S**ynchronized-pump **M**anagement **A**lgorithms for **R**eliable **T**herapies) platform [4].

## V. Conclusion

We designed, built, and tested a novel compact microfluidic spectroscopy method for continuously measuring in real time the concentration of multiple solutes flowing together in a shared, continuous stream driven by infusion pump. We also developed a standalone MATLAB application that serves as central hub for conveniently controlling all aspects of the microscopic spectroscopy platform. Leveraging these advances, we show that our system can reliably and repeatably measure the concentration of between two model light absorbing dyes, which served as convenient surrogates for solutes such as clinically relevant medications used in anesthesia and critical care settings. The ability to perform multiplexed time-resolved concentration measurements with high temporal resolution is expected to be broadly important in biomedicine and industrial settings, and play a critical role in facilitating the translation of emerging technologies for drug infusion management [4].

Despite its promise, the proposed methodology has several limitations. First, this technique requires strongly light adsorbing solutes for making concentration measurements, while many practical solutes of interest such as drugs often have very weak light absorption. In such cases, light absorbing dyes will need to be mixed in with the solute of interest, indirectly measure the latter's concentration. Second, we anticipate that the methods will be scalable to measuring the concentrations of 3 or more solutes in a multi-component fluid, either for diagnostics or for technology development. However, measurement capability will depend upon whether the different solutes have absorption spectra sufficiently discrete (minimal overlap) to allow concentration determinations for individual components. Third, we only tested clinical syringe pump based infusion systems. Although we anticipate similar overall findings with different types of pumps and delivery components, this remains to be formally evaluated. Finally, we did not test a wide range of fluid flows. It is possible that turbulence or other physical interaction between two or more fluid flows entering the common fluid pathway at widely different rates would impact uniformity of solute concentration at a discrete time point.

## Data Availability

Data will be made available on request.

## Supplementary Materials

**MATLAB APP LINK:** https://github.com/andrea199/MatlabSpectroscopy
