# Supplementary material for: A Method for Temporally Resolved Continuous Inline Measurement of Multiple Solute Concentrations With Microfluidic Spectroscopy
Source: IEEE Open J Eng Med Biol. 2025 Mar 28;6:442–9. doi: 10.1109/OJEMB.2025.3555807 (PMC12251075; doi:10.1109/OJEMB.2025.3555807)
Supplement: Supplementary Materials [file supp1-3555807.pdf]

Supplementary Materials

A method for temporally resolved continuous inline measurement of multiple solute concentrations with microfluidic spectroscopy

A. L. H. S. Detry[1,2*], V. Chandran Suja[4,5*], N. M. Sims[2†], R. A. Peterfreund[2†], and D. E. Arney[2†] [1] Department of Chemical, Materials and Industrial Production Engineering, University of Naples Federico II, 80125 Naples, Italy
[2] Massachusetts General Hospital, Department of Anesthesia, Critical Care and Pain Medicine, 55 Fruit Street, Boston, MA 02114, USA
[4] School of Engineering and Applied Sciences, Harvard University, MA - 02134, USA
[5] Wyss Institute for Biologically Inspired Engineering, 52 Oxford St, Cambridge, MA 02138, USA
CORRESPONDING AUTHOR: A. L. H. S. Detry (e-mail: andrea.detry@unina.it)
∗ These authors contributed equally to this work
† Co-Senior authors

I. MATLAB App

To conveniently monitor and control all aspects of our experimental platform we developed a stand alone MATLAB App (Fig. S1). The App's Graphical User Interface (GUI) features an intuitive interface with various functionalities such as controlling the spectrophotometer operation, syringe pump control, real-time concentration measurement, data logging, and monitoring the analytical balance. The following is a description of its features:

- The spectrophotometer section of the app interface displays the acquired spectrum in real-time (Fig. S1a). Users can customize the spectrophotometer's data acquisition to their experiment's requirements by entering parameters such as integration time, bin wavelength, bin half-width, and acquisition interval. The Real Time Intensity plots display the spectral intensity at user defined bin wavelengths, in this case at 424 nm and 631 nm (Fig. S1b,c). The start and stop acquisition buttons enable on-demand data collection, while status indicators provide immediate feedback on the system's operation.
- The Pump Operation section of the application is designed for the management of the infusion pumps. Users can set the steady state and maximum flow rates for each pump and initiate their operation. The interface displays the actual flow rates, along with controls to start or stop each pump individually.
- The Real-Time Concentration area includes two dynamic plots that activate upon user command, displaying the concentration of fluids in the system as calculated from the spectrophotometer data (Fig. S1d,e). These plots are updated in real-time, reflecting the changing concentrations based on the calibration equations provided in Section II.
- The section on Data Logging is intended for recording experimental data. Users can connect to external hardware, such as an Arduino, to control the stopcock assembly (Section Materials and Method/Stopcock) and specify the file for data storage. Experiments can be initiated and terminated with dedicated buttons. To appropriately normalize the intensities (Fig. S1b,c) and obtained concentrations (Fig. S1d,e), it is necessary to establish the baseline intensities for pure saline (zero dye concentrations) and pure dye solutions (max dye concentrations). This can also be accomplished via functionality provided in the Data Logger section, prior to starting an experiment.
- The Scale Monitoring section provides a time resolved visualization of the total infused fluid mass. This feature is crucial for experiments that require flow rate measurements. When activated, the plot can be viewed in real-time, and the value at each time point is saved in a log file.

II. Calibration

In order to recover the concentrations of Tartrazine (C_{TZ}) and Erioglaucine Blue (C_{EG}) from the spectrophotometer data, we calibrate the spectral intensities for the two dyes with a series of mixtures with known dye concentrations. The calibration graph for Erioglaucine Blue is illustrated in Fig S2b. Solid circles indicate measurements at 424 nm, and open circles denote the data at 631 nm. The solid curves correspond to single exponential fits while dashed lines correspond to linear fits. As expected, a rise in Erioglaucine Blue concentration leads to a decrease in normalized light transmission intensity, aligning with the Beer-Lambert principle that absorbance is proportional to substance concentration. Analogously, Tartrazine's calibration is determined by its effect on light transmission intensity across concentrations at these wavelengths, as shown in Fig S2c. Appropriate numerical fits to the calibration data for both the dyes yield Eqns.1 - 4.

$$I_{631EG} = \exp(-0.1563 \times C_{EG}) \times 1.015 \quad (1)$$
$$I_{424EG} = -0.006415 \times C_{EG} + 1.001 \quad (2)$$
$$I_{424TZ} = \exp(-0.05342 \times C_{TZ}) \times 1.027 \quad (3)$$
$$I_{631TZ} = 5.936e-06 \times C_{TZ} + 0.9994 \quad (4)$$

To recover the concentrations C_{TZ} and C_{EG} from the measured intensities (I_{631}) and (I_{424}) in an arbitrary dye mixture,

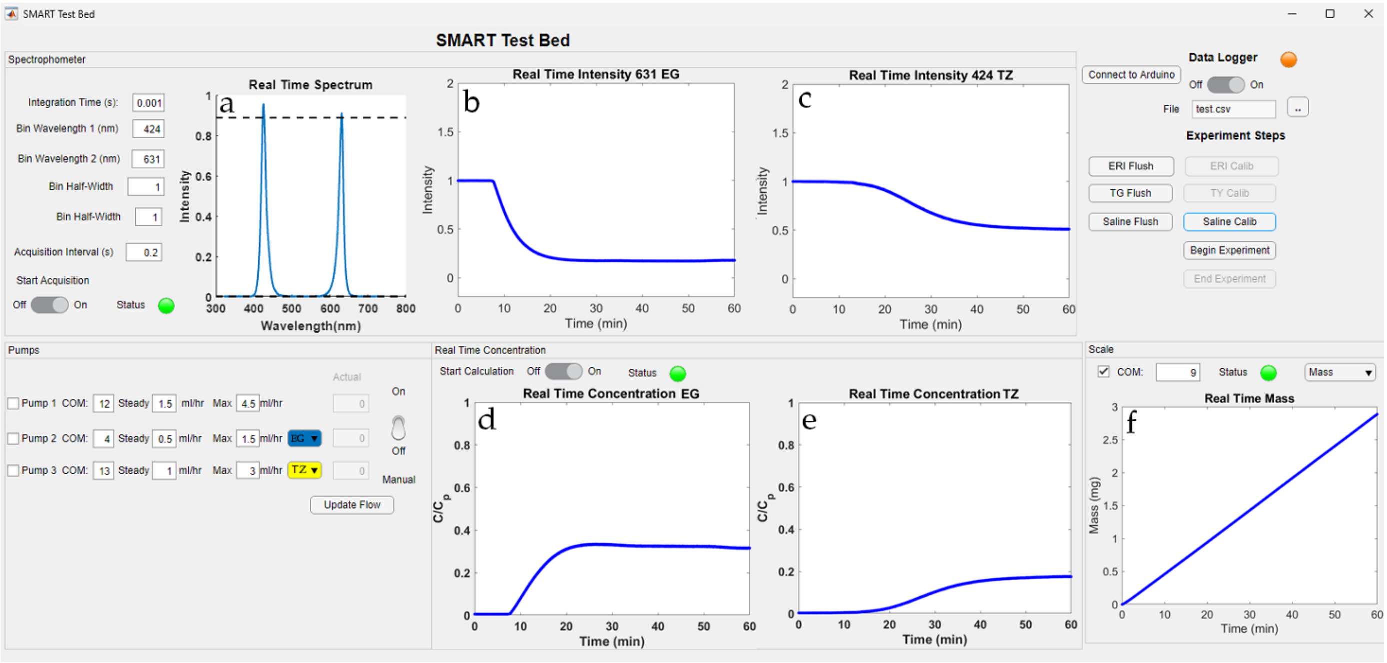

Fig. S1. Interface of the MATLAB Application with representative data expected from a typical experiment. Panel (a) displays the real-time spectrum. Panels (b) and (c) show the real-time intensity plots for Erioglaucine (EG) at 631 nm, the absorption peak for EG (b) and for Tartrazine (TZ) at 424 nm, the absorption peak for TZ (c). The decrease in intensity observed in these panels corresponds to the increase in dye concentration. Panels (d) and (e) show the real-time concentration (C) of Erioglaucine and Tartrazine, respectively, normalized as C/C_0, where C_0 is the concentration of the dye in the syringe (or equivalently the maximum possible concentration). Panel (f) presents the real-time mass measurement from the scale. The spectrophotometer section (top left), the pumps section (bottom left), the real-time concentration graphs (bottom center), the Data Logger section (top right), and the analytical balance/scale section (bottom right) complete the interface layout.

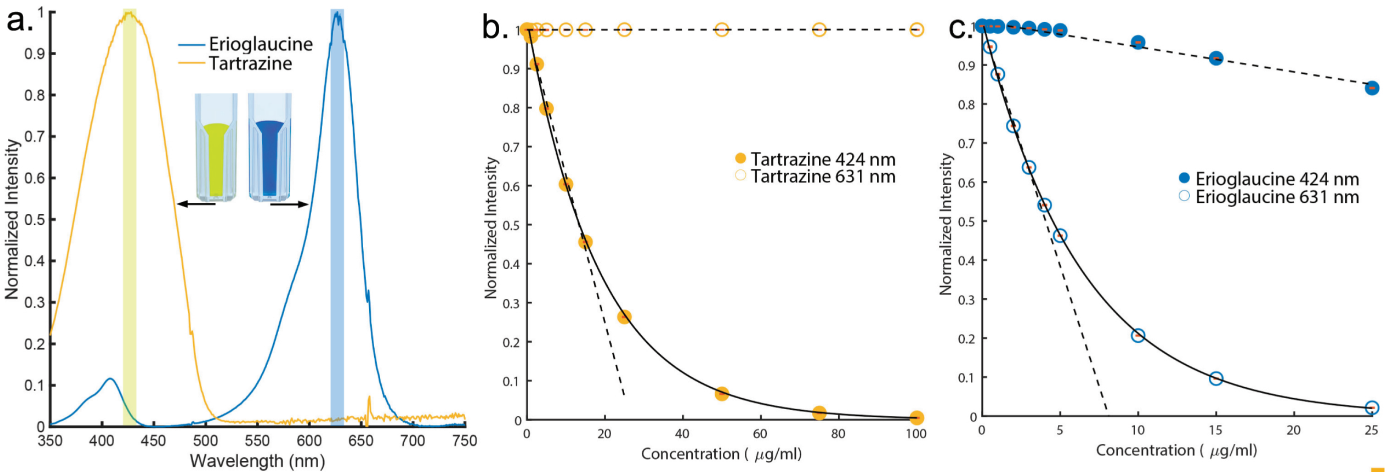

Fig. S2. **a.** Absorption spectrum of Erioglaucine Blue and Tartrazine. Concentration versus normalized transmission intensity calibration curves for Erioglaucine Blue **b.** and Tartrazine **c.** Solid curves correspond to single exponential fits while dashed lines correspond to linear fits.

it's necessary to solve a pair of coupled nonlinear equations,

$$\begin{cases} F_1 = I_{631} - I_{631T} - I_{631E} + 1 \\ F_2 = I_{424} - I_{424T} - I_{424E} + 1 \end{cases} \quad (5)$$

We employ MATLAB's **fsolve** function for numerically solving these equations, for near real-time determination of the true concentrations (C_{TZ}) and (C_{EZ}).
